# Decoding the Enigma: Translation Termination in Human Mitochondria

**DOI:** 10.1093/hmg/ddae032

**Published:** 2024-05-23

**Authors:** Annika Krüger, Daria Kovalchuk, Dmitrii Shiriaev, Joanna Rorbach

**Affiliations:** Department of Medical Biochemistry and Biophysics, Division of Molecular Metabolism, Karolinska Institutet, Solnavägen 9, Solna 171 65, Sweden; Department of Medical Biochemistry and Biophysics, Division of Molecular Metabolism, Karolinska Institutet, Solnavägen 9, Solna 171 65, Sweden; Department of Medical Biochemistry and Biophysics, Division of Molecular Metabolism, Karolinska Institutet, Solnavägen 9, Solna 171 65, Sweden; Department of Medical Biochemistry and Biophysics, Division of Molecular Metabolism, Karolinska Institutet, Solnavägen 9, Solna 171 65, Sweden

**Keywords:** Mitochondrial translation, mitochondrial translation termination factors, mitoribosomes

## Abstract

Mitochondrial translation is a complex process responsible for the synthesis of essential proteins involved in oxidative phosphorylation, a fundamental pathway for cellular energy production. Central to this process is the termination phase, where dedicated factors play a pivotal role in ensuring accurate and timely protein production. This review provides a comprehensive overview of the current understanding of translation termination in human mitochondria, emphasizing structural features and molecular functions of two mitochondrial termination factors mtRF1 and mtRF1a.

## Introduction

**Figure 1:**
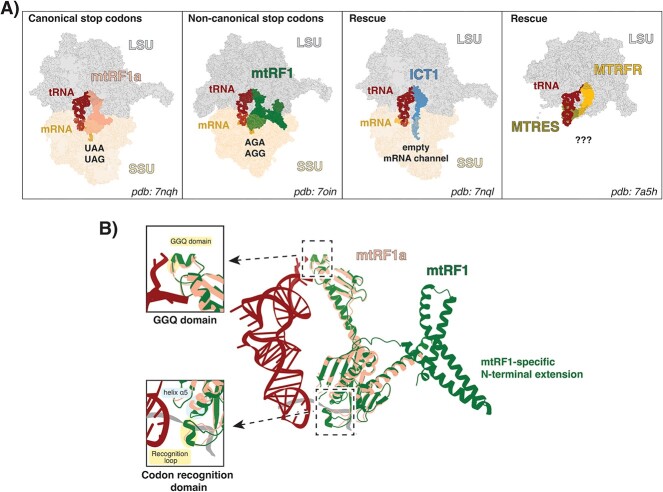
Cryo-EM structures of the mitochondrial release factor family. (A) Mitochondrial release factors mtRF1a, mtRF1, ICT1, and MTRFR in their mode of action at the mitoribosome. MtRF1a recognizes the canonical stop codons UAA/UAG, mtRF1 recognizes the non-canonical stop codons AGA/AGG, ICT1 can bind to mitoribosomes with empty mRNA channels, and MTRFR binds in complex with MTRES to the LSU. The exact substrate of MTRFR is still unknown. (B) Alignment of the cryo-EM structures of mtRF1a (pdb: 7nqh) and mtRF1 (pdb: 7oin). MtRF1 exhibits an N-terminal extension compared to mtRF1a. The left panel shows zoomed-in views of the GGQ domain and the codon recognition domain. While the GGQ domain is conserved between both release factors, the codon recognition domain shows substantial differences regarding the codon recognition loop and helix α5.

The termination phase of translation plays a pivotal role in the accurate synthesis of proteins. Termination in mitochondria, similar to the prokaryotic and eukaryotic translation systems, is coordinated by critical protein-binding events. A key step presents the recognition of a stop codon by specific release factors (RFs), which subsequently mediate release of the newly translated protein. However, some aspects of translation termination are unique to mitochondria and the termination process is tailored to accommodate these evolutionary differences. While most prokaryotic and eukaryotic translation systems terminate translation at the canonical stop codons UAA, UAG and UGA, the mitochondrial genome presents deviations from the standard genetic code. In human mitochondria, UGA encodes tryptophan instead of a stop codon, and two additional stop codons, AGA and AGG,have emerged from the reassigned arginine codons. These non-canonical stop codons are found at the end of *COX1 *and *ND6* open reading frames (ORFs), respectively [1]. Refined bioinformatic approaches have identified four putative translation termination factors in mitochondria—mtRF1a, mtRF1, ICT1 (mL62) and MTRFR—based on their homology to known bacterial RFs and the conservation of the essential catalytic GGQ motif, which is crucial to facilitate peptide hydrolysis [2–8]. Both mtRF1a and mtRF1 contain all domains found in canonical RFs, whereas MTRFR and ICT1 lack the codon recognition domain and therefore have been proposed to rescue stalled mitoribosomes. Molecular details of ICT1 and MTRFR function were revealed by recent cryo-EM structures, which demonstrate that ICT1 recognizes mitoribosomes with empty A-sites (e.g. truncated mRNAs) [9] and MTRFR binds to split large subunit (LSU) moieties [10] (Fig. 1A). The function of mtRF1a as a canonical RF, recognizing UAA and UAG stop codons, was revealed more than 15 years ago [3, 4]. In contrast, the role of mtRF1 in non-canonical stop-codon recognition has been under debate for many years and has only been clarified recently. The combination of mitoribosome profiling data and an *in vitro* translation termination assay [11], together with the cryo-EM structure of mtRF1 in a mitoribosome termination complex [12] provided strong evidence that mtRF1 indeed functions as a classical RF recognizing the non-canonical stop codons AGA and AGG in mitochondria.

Herein, we provide an updated overview of the translation termination process at canonical and non-canonical stop-codons in mammalian mitochondria. We highlight the most important discoveries, discuss the molecular similarities and differences between mtRF1a and mtRF1, and present the evolutionary context of the function of both RFs, followed by a perspective of the knowledge gaps and open questions.

## Historical perspective

The presence of a mitochondrial RF was first detected in 1987 in crude mitochondrial extracts from rats [13]. Even though its release activity towards UAA and UAG stop codons could already be demonstrated back then, it took 20 more years to identify the protein responsible for these observations—mtRF1a [3, 4]. Upon the discovery of mtRF1a and its role in canonical translation termination, questions regarding the possible role of mtRF1 emerged. MtRF1 was discovered in 1989 via a database search [2], but its function has remained elusive. As the human mitochondrial transcriptome possesses both canonical UAA/UAG and non-canonical AGA/AGG stop codons at the end of ORFs, a role of mtRF1 as the second mitochondrial RF recognizing the latter two codons was plausible. This theory was supported by bioinformatic and phylogenic analysis demonstrating the high homology between mtRF1a and mtRF1 and the coincidence of the origin of mtRF1 and AGA/AGG codons at the root of the vertebrate lineage [8, 14]. However, as several experimental attempts to show the release activity of mtRF1 at AGA/AGG codons failed [3, 4, 9, 15], the debate about the actual function of mtRF1 and the release mechanism at AGA/AGG codons continued. For example, it was suggested that ICT1, rather than mtRF1, would mediate release at non-canonical stop codons [15]. Another interesting study has put forth the intriguing idea that mitoribosomes would terminate translation at non-canonical stop codons via a −1 frameshift that would place the canonical stop codons UAA into the A-site [16]. Yet, none of these hypotheses were supported by direct experimental evidence. After more than 10 years of debate, new efforts to reveal the function of mtRF1 finally succeeded. The initial evidence supporting the role of mtRF1 in COX1 termination, but distinct from ND6, came from a biochemical study analysing the cellular consequences of the loss of mtRF1 [17]. Shortly after, the direct involvement of mtRF1 in releasing at both non-canonical stop codons could be demonstrated via ribosome profiling as well as an advanced *in vitro* termination assay [11]. Finally, these data were confirmed by structural analysis via cryo-EM revealing the binding mechanism of mtRF1 to AGA/AGG codons [12]. 

## Molecular mechanism and structural aspects of mtRF1a and mtRF1 stop codon recognition

When the mitoribosome reaches the end of an ORF, translation is terminated by an RF, which recognizes a stop codon lacking a cognate tRNA and induces the release of the nascent polypeptide. Precise differentiation between stop and sense codons is mediated via the codon recognition domain of RFs and is crucial to selectively release the fully developed polypeptides [18]. Bacteria possess two RFs, RF1 and RF2, which read three stop codons in a semi-specific manner. While UAA stop codons are read by both RFs, UAG is specifically read by RF1 and UGA by RF2. The codon recognition mechanism, however, is highly conserved between both RFs and involves direct interactions of their amino acid side chains with the mRNA bases [19]. In contrast, recent cryo-EM structures of the two mitochondrial RFs, mtRF1a and mtRF1, revealed very different binding modes of the codon recognition domains. While mtRF1a preserved the classical recognition elements from the bacterial system recognizing UAG and UAA [9], mtRF1 exhibits several insertions within the codon recognition domain resulting in a unique binding mechanism of the non-canonical stop-codons AGA and AGG [12]. The extended codon recognition loop of mtRF1 forms a short α-helix (Fig. 1B), which instead of interacting with the stop codon, pushes the two first bases of the stop codon towards the head region of the ribosome small subunit (SSU). This reorientation is further stabilized by an extended tip of helix α5 (Fig. 1B). As a result, the non-canonical stop codons are placed in such a way, that they can be specifically recognized by mtRF1 via a network of interactions including residues of the RF, neighbouring bases of the mRNA and residues of the rRNA of the SSU. This unique binding mechanism of mtRF1 not only made predictions of its binding mode by computational modelling challenging but also probably hampered its study via *in vitro* termination assays, which routinely use bacterial instead of mitochondrial components. Therefore, it is not surprising that the function of mtRF1 as a classical RF has only been deciphered recently.

The hydrolysis mechanism of the peptide-tRNA bond during translation termination is highly conserved among species and involves the GGQ motif within the peptidyl-tRNA hydrolase (PTH) domain of RFs. Typically, RFs undergo structural rearrangements after stop codon recognition, which places their catalytic PTH domain into the PTC to assist peptidyl-tRNA hydrolysis. Even though both mitochondrial RFs contain the highly conserved GGQ domain (Fig. 1B), and cryo-EM data of the termination complexes suggest a similar mechanism, the structural rearrangement still needs to be demonstrated experimentally. Not only the GGQ motif but also its methylation at the Q residue is highly conserved. This post-translational modification was shown to enhance the catalytic activity in peptide release [20, 21] and has been suggested to be important for controlled conformational changes of the RF to recognize the stop codon [22].

Another difference between the two mitochondrial RFs, mtRF1a and mtRF1, represents the N-terminal domain. In contrast to mtRF1a, mtRF1 contains an N-terminal extension with several positively charged amino acids (Fig. 1B). The recent cryo-EM structure of mtRF1 bound to the non-canonical stop-codons demonstrated that this extension protrudes into a pocket below the L7/L12 stalk of the LSU, which might help to stabilize the binding of mtRF1 to the mitoribosome.

## Stop codon usage in mitochondria in vertebrates

**Figure 2:**
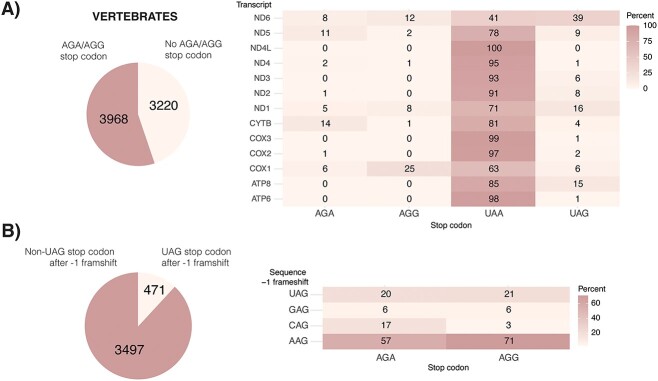
Stop codon usage and context in mitochondria in vertebrates. (A) *Left panel:* Number of vertebrates exhibiting AGA/AGG stop codons within their ORFs. *Right panel:* Percentage of stop codon usage in individual transcripts. Stop codons having < 0.5% contribution for each transcript are not shown. UAA presents the most common stop codon in all transcripts. (B) *Left panel:* Number of vertebrates having uracil at the −1 position of AGA/AGG stop codons. *Right panel:* Codon context in vertebrates at the −1 position of AGA/AGG stop codons. AAG presents the most common codon after a −1 frameshift. A set of annotated mitochondrial genomes (16 405 records) was obtained from NCBI organelle genome collection (https://ftp.ncbi.nlm.nih.gov/refseq/release/mitochondrion/, downloaded on 31 October 2023) and filtered to 7206 genome records related to vertebrates. Out of those, records of 6 species, containing two genome records, and 6 species containing no reference sequences, were discarded. For the resulting 7188 records, the last three nucleotides of each ORF were analysed, as well as the 5′-adjacent nucleotide. Python code used for extraction and analysis is deposited in the repository: https://github.com/dm-shr/genome_analysis_stop_codons.

MtRF1a is the most widely distributed mitochondrial RF among organisms [8]. It evolved from an alphaproteobacterial ancestor like the organelle itself. In contrast, mtRF1 is a vertebrate-specific mitochondrial RF [3], which arose from a gene duplication event of mtRF1 [14]. Its origin at the root of the vertebrate lineage coincides with the appearance of AGG/AGA stop codons [8], supporting the function of mtRF1 as RF recognizing these non-canonical stop codons. The usage of AGA/AGG stop codons varies between different vertebrate species and both codons can be found in almost any mitochondrial gene, such as *ND1*, *ND2*, *ND4*, *ND5*, *ND6*, *COX1*, *COX2*, and *CYTB* (Fig. 2A). The highest occurrence of AGG shows *COX1* with 25% of all vertebrates with annotated mitochondrial genomes having this codon at the end of the ORF. The highest occurrence of AGA can be found at the end of *CYTB* with 14% of all vertebrates having this codon. Despite the presence of mtRF1 in all vertebrates, 44% of vertebrates do not harbour AGA/AGG codons at the end of mitochondrial ORFs, raising the question of the function of mtRF1 in these organisms. Potential reasons for the preservation of mtRF1 in these organisms are discussed in the section “Ongoing question”.

Before the function of mtRF1 as classical RF could be demonstrated, an alternative mechanism for translation termination at non-canonical stop codons was suggested—the so-called “frameshift theory” [16]. The theory implies that instead of recognizing AGA/AGG as stop codons, the mitoribosome would step one codon back, placing the canonical UAG stop codon in the A-site, which then subsequently can be recognized by mtRF1a. Even though this theory is plausible for translation termination at non-canonical stop codons in some organisms, the preceding uracil is not evolutionary conserved and 3497 vertebrate species do not show uracil in the consecutive upstream position (Fig. 2B). Instead, adenine is most prevalent at this position in vertebrates (Fig. 2B), which would result in lysine rather than a stop codon. Consequently, the frameshift theory is unlikely to be an evolutionarily conserved mechanism to terminate translation at non-canonical stop codons.

## Ongoing questions

Even though recent studies successfully revealed the function of mtRF1 as classical RF, several research questions remain open.

One puzzling result presents the cellular phenotype of *mtRF1* KO HEK cells [11, 17]. While the absence of mtRF1 causes a decrease in COX1 protein levels, ND6 protein levels are not affected. As data from ribosome profiling experiments clearly showed mitoribosome stalling events on both transcripts in *mtRF1* KO cells [11], consecutive downstream effects must lead to these differences. While multiple reasons could explain these different outcomes, we will only discuss the two most likely ones.

On the one hand, differences in the rescue process on both transcripts, resolving the stalled mitoribosome and releasing the newly translated protein, could explain the distinct downstream effects. So far, there is little information on rescue processes in mitochondria, yet two RFs have been identified to be involved: ICT1 and MTRFR. As ICT1 is likely resolving mitoribosomes on truncated transcripts, mitoribosomes stalled on stop codons do not represent ideal targets for ICT1. Unlike ICT1, the context of MTRFR’s function as a rescue factor has not been identified yet, but its involvement in rescuing mitoribosomes upon loss of mtRF1 has been suggested [17]. As this hypothesis could, however, not be confirmed in a second study [11], further data is needed for clarification, which also might help to understand the transcript-specific downstream effects.

On the other hand, it is known from other translation systems that the read-through of stop codons by near-cognate tRNAs is a competing process for translation termination [23]. The likelihood of read-through is determined by the availability of RF, concentration of near-cognate tRNAs, and is also dependent on the codon context [24, 25]. Consequently, the loss of RFs greatly increases the probability of ribosomes reading through a stop codon [23, 26]. Up to date, there is no information on read-through events in mitochondria. Still, it can be speculated that the loss of mtRF1 significantly increases read-through events on *COX1* and *ND6* transcripts. As both transcripts harbour very different 3′ UTRs (e.g. short vs. long UTR, polyA- vs. no polyA-tail), read-through events might trigger very different downstream events. So far, this hypothesis is only based on theory and therefore must be confirmed experimentally in future studies.

Another open question concerns the role of mtRF1 in organisms lacking AGA/AGG codons within the ORF. On the one hand, this observation suggests that mtRF1 possesses a function independent of its RF activity, which causes its evolutionary preservation. Several mitochondrial translation factors have been proposed to possess dual functions within this organelle including ICT1 [6], mtEF-Tu [27], and GTPBP6 [28]. However, as the loss of mtRF1 seems to specifically impact COX1 translation and no other pathways [11, 17], there are currently no hints towards a second role of mtRF1. On the other hand, the preservation of mtRF1 might function as a backup mechanism in these species. Several events, such as read-through, mitoribosome frameshifting, misprocessing, or DNA decoding errors can disrupt the canonical reading frame, which could lead to AGA/AGG codons within the A-site of mitoribosomes. Consequently, the presence of mtRF1 would help resolve these mitoribosomes and recycle them for further translation rounds. Interestingly, a previous study on the plant species *Dactylodes fasciculatum* has made similar observations. While the stop codon UGA is absent in the mitochondrial genome, the UGA-specific RF2 factor is still preserved [29].

In conclusion, these ongoing questions demonstrate the need to further explore the translation process in mitochondria. While we have a good understanding of the process of classical translation termination, we are still at the beginning of understanding more complex mechanisms occurring upon errors in protein synthesis. Combining state-of-the-art techniques, such as proteomics, transcriptomics, and cryo-EM, together with the development of new model systems will likely push forward this new research field in the following years.
